# Assessing the effects of multiple markers in genetic association studies

**DOI:** 10.3389/fgene.2015.00066

**Published:** 2015-02-24

**Authors:** Xuefeng Wang, Joanna M. Biernacka

**Affiliations:** ^1^Department of Preventive Medicine, Stony Brook UniversityStony Brook, NY, USA; ^2^Department of Health Sciences Research, Mayo ClinicRochester, MN, USA

**Keywords:** genetic association studies, multiple-marker, family-based designs, gene-gene interactions, genetic risk prediction

It is believed that human diseases and their underlying causal pathways involve a complex interplay between multiple genetic and environmental risk factors. The search for relevant genetic variants, which typically have very small individual effects, is challenging. While typically genetic association studies involve testing for association between the phenotype and each individual genetic variant (e.g., single nucleotide polymorphism, SNP), it is widely recognized that jointly assessing the effects of multiple genetic markers can increase power or provide the opportunity to test different hypotheses than those addressed by single-maker analyses. Various statistical approaches that enable the joint use of data from multiple genetic markers are under development. This eBook contains a selection of manuscripts dealing with various aspects of assessing the effects of multiple markers in genetic association studies, including: gene or region-based association testing approaches, particularly for rare variant analysis; study of gene-gene and gene-environment interactions; model selection and prediction using high-dimensional genomic data.

Multi-marker tests of association can be used to maximize power to detect association at the gene or regional level. The study by Burkett et al. (2013) included in this issue considered an approach for a multi-marker regional test. In particular, they demonstrate how gene genealogies estimated from haplotype data can be used to find disease-predisposing genetic variants and propose a tree-based test of association based on assessing haplotype similarity of cases versus controls. Noting that genotype correlations within an LD block asymptotically lead to a multivariate normal distribution for score test statistics, Taub et al. (2013) developed a set of weights for markers to maximize power of multi-marker association tests, and found that a method previously proposed by Conneely and Boehnke (2007) is a practical and powerful method for a range of scenarios.

Region or gene-level tests are particularly useful for rare variant analysis, because the power of typical single-marker association tests is very low for rare variants. Several manuscripts in this issue focused on rare variant analysis. Notably, Thomas et al. (2013) provide a comprehensive review on methods and analysis strategies for next generation sequencing studies, focusing on two major types of study designs: two-phase design for subject subsampling, and family-based design for variant prioritizing. Various issues are investigated using simulations and preliminary data from two studies, providing valuable guidance for sequencing study design in both pedigrees and unrelated samples. Stewart and Cerise (2013) also suggested harnessing the power of family based designs in association tests and prioritization of genetic variants/regions—with a particular interest in SNPs with MAFs between 0.03 and 0.12. They proposed a novel non-parametric association test, which can accommodate large families and case-control data. It is expected that such method will better inform the design of follow-up sequencing efforts. Yoo et al. (2013) proposed a multiple regression method for gene-level association tests for quantitative traits, using both common and rare variants, and found that their approach applied to both common and rare variants provided a robust and powerful alternative to analyzing the common and rare SNPs separately. Also in this issue, Xu et al. (2014) explored the potential utility of stratified false discovery rate for region-based association tests for rare variant data, concluding that their simulations demonstrated low power for window-based tests, and that the estimated FDR values tended to be much smaller than the true FDRs, likely at least partially due to long-range linkage disequilibrium. They suggested that use of external annotation information may improve power, but warned that sample sizes in current sequencing studies will not enable detection of many causal variants of realistic effect sizes. Finally, Cook et al. (2014) explored the impact of genotyping errors on rare variant association tests. They conclude that different types of errors in SNP genotyping can lead to inflated type I error rate and decreased power, while certain rare variant tests and study designs may be more robust to genotype errors.

Investigation of gene-gene interactions also involves multi-marker analyses. Six manuscripts in this eBook addressed gene-gene interactions, or the related topic of gene-environment interactions. Lee et al. (2013) and Sung et al. (2014) investigated methods for the analysis of interactions in family data. Chen and Guo, and Millstein discussed potential solutions to the challenge of high dimensionality in interaction studies. Simino et al. (2013) and de las Fuentes et al. (2013) report their analyses of large-scale epidemiologic cohorts, studying gene-alcohol and gene-drug interactions, respectively. These papers highlighted two key challenges in the current applications of methods for detecting interaction effects. First, computational burden can become prohibitive when interactions are investigated in genome-wide data. Chen and Guo (2013) explored the possibility of overcoming this constraint through the use of processor graphics cards (GPU), while in the analysis of longitudinal family data, Sung et al. (2014) proposed using a computationally less intensive method based on the hierarchical linear model (HLM). Second, it is well-known that interaction analyses require very large sample sizes, and real data analyses often fail to achieve genome-wide significance (as shown in de las Fuentes et al., 2013). It remains an open question to what extent top-ranking (but non-significant) findings are informative in prioritizing genes for future studies.

Genetic risk prediction is an important area of growing interest, which is ideally performed in a multi-marker framework. Although risk prediction is recognized as an important goal of human genetics research, efficient statistical methods are still not well-established, and few studies have demonstrated successful use of genetic risk prediction for complex traits. In this issue, Che and Motsinger-Reif (2013) expand on their earlier work comparing simple genetic risk scores with weighted risk scores, demonstrating that the weighted methods outperform simple count risk scores in general, including more complex situations involving interactions or presence of linkage disequilibrium. Using longitudinal data, Wineinger et al. (2013) applied methods including n-fold cross-validation procedures to generate lipid genomic prediction models based on previously reported genetic markers, which led to improved prediction over non-genetic risk models.

Achieving both computational (and cost) efficiency in addition to statistical efficiency is a key challenge in multi-marker testing. The contribution by Millstein and Volfson (2013) provides a novel method (and an R package “fdrci”) where permutations can be used to estimate FDR including confidence intervals in a non-parametric manner, which can account for dependencies among tests and is computationally parsimonious.

In summary, the articles in this *Frontiers in Genetics* Research Topic have described and applied various approaches that aim to better exploit currently available data with appropriate statistical approaches, and discussed the technical challenges and computational issues that remain in practical data analysis. Application of powerful novel analytical methods such as those described in this Research Topic is a key factor enabling progress in complex human disease research.

## Conflict of interest statement

The authors declare that the research was conducted in the absence of any commercial or financial relationships that could be construed as a potential conflict of interest.
